# Euglycemic DKA in a DPP-4 inhibitor patient following traumatic brain injury: A complex ICU glucose management challenge

**DOI:** 10.1097/MD.0000000000043562

**Published:** 2025-07-25

**Authors:** Isam Shammas, Mohammed Seyaj, Kenana Altell, Razan Sobeih, Lara Asafrah, Malek Burqan, Mamoun Abuhalawa, Mohammed Shahateet, Saeed Itkaidek

**Affiliations:** a Department of Surgical ICU, Al-Ahli Hospital, Hebron, State of Palestine; b Department of Medicine, Hebron University, Hebron, State of Palestine; c Department of Neurosurgery, Al-Ahli Hospital, Hebron, State of Palestine.

**Keywords:** DPP-4 inhibitors, euglycemic diabetic ketoacidosis, ICU, TBI

## Abstract

**Rationale:**

Euglycemic diabetic ketoacidosis (EuDKA) is a rare variant of diabetic ketoacidosis (DKA), characterized by normal or near-normal glucose levels despite severe metabolic acidosis and ketosis. This condition poses a diagnostic challenge as it mimics DKA without the hallmark hyperglycemia. Recent studies have suggested an association between EuDKA and newer antidiabetic medications, including dipeptidyl peptidase-4 (DPP-4) inhibitors.

**Patient concern:**

A 57-year-old male with a history of type 2 diabetes, hypertension, ischemic heart disease, and rheumatoid arthritis developed EuDKA following a traumatic brain injury. Despite a blood glucose of 134 mg/dL, he exhibited classic features of DKA, including severe metabolic acidosis, low bicarbonate levels, an elevated anion gap, and positive urinary ketones.

**Diagnosis:**

Despite the blood glucose level being within normal range, the patient’s clinical presentation, including severe acidosis (pH 7.16), low bicarbonate (2.8 mmol/L), elevated anion gap (24.9 mmol/L), and urinary ketones, pointed to EuDKA. The patient’s use of Sitagliptin, a DPP-4 inhibitor, was identified as a likely precipitating factor.

**Interventions:**

Timely interventions, including fluid resuscitation, insulin therapy, and electrolyte correction, were promptly administered to manage the patient’s metabolic abnormalities.

**Outcome:**

After receiving intensive treatment, the patient made a full recovery with normalization of metabolic parameters and no long-term complications.

**Lessons:**

This case emphasizes the importance of maintaining a high index of suspicion for EuDKA in trauma patients, particularly those on DPP-4 inhibitors. Early recognition and prompt intervention are crucial to preventing complications and ensuring positive outcomes. Further research is needed to explore the mechanisms linking DPP-4 inhibitors with EuDKA, especially in the context of metabolic stress like traumatic brain injury.

## 1. Introduction

Diabetic ketoacidosis (DKA) is a life-threatening complication of diabetes mellitus, primarily caused by an absolute or relative insulin deficiency. This deficiency prevents the body from using glucose for energy, resulting in fat breakdown, which in turn leads to the excessive production of ketone bodies. These ketones cause significant metabolic acidosis, in which the blood becomes too acidic. The American Diabetes Association defines the diagnostic criteria for DKA as a plasma glucose level >250 mg/dL, an arterial pH below 7.30, and a serum bicarbonate level of 18 mEq/L or less. Prompt recognition and treatment are crucial in preventing severe complications such as cerebral edema, coma, and death.^[1]^

Euglycemic Diabetic Ketoacidosis (EuDKA) represents an intriguing variant of this condition. While it shares many of the same features as classic DKA, such as low bicarbonate levels and ketoacidosis, it is distinguished by normal or near-normal blood glucose levels. This unique presentation makes it significantly more difficult to diagnose and leads to frequent misdiagnosis. EuDKA was once considered a rare phenomenon but has become more commonly recognized, especially with the growing use of newer antidiabetic medications, particularly sodium-glucose cotransporter-2 (SGLT2) inhibitors, as well as recent insulin use, reduced caloric intake, heavy alcohol consumption, chronic liver disease, and glycogen storage disorders.^[2]^

One of the main challenges in identifying EuDKA is the absence of hyperglycemia, the hallmark of typical DKA, so, this condition may go unnoticed by healthcare providers who expect elevated glucose levels as a diagnostic indicator.^[3]^

EuDKA is most commonly associated with SGLT2 inhibitors, which lower blood glucose through their effects on the kidneys.^[2]^ However, recent studies have also highlighted the potential role of other medications, including dipeptidyl peptidase-4 (DPP-4) inhibitors. DPP-4 inhibitors, such as Sitagliptin, Saxagliptin, and Linagliptin, have been used since 2006 and are widely prescribed for managing type 2 diabetes mellitus. These drugs do not directly lower blood glucose; instead, they protect incretin hormones, which help regulate blood sugar levels. This glucose-dependent mechanism reduces the risk of hypoglycemia, a common side effect of many antidiabetic medications. However, DPP-4 inhibitors can influence glucagon levels, which may, in some cases, create a metabolic environment that favors ketogenesis. When combined with other risk factors, such as fasting, infections, or dehydration, the likelihood of EuDKA increases. Clinicians must remain vigilant in recognizing the potential for EuDKA, especially in patients taking DPP-4 or SGLT2 inhibitors. Both drug classes can indirectly promote ketone production, particularly in vulnerable patients, making close monitoring crucial to prevent serious complications.^[1,4]^

Overall, EuDKA requires heightened awareness among healthcare providers, particularly with the growing use of newer antidiabetic therapies. Timely identification and appropriate treatment are essential to prevent adverse outcomes in patients who might otherwise be misdiagnosed or undiagnosed due to the absence of classic hyperglycemia.

## 2. Case presentation

**Figure 1.**
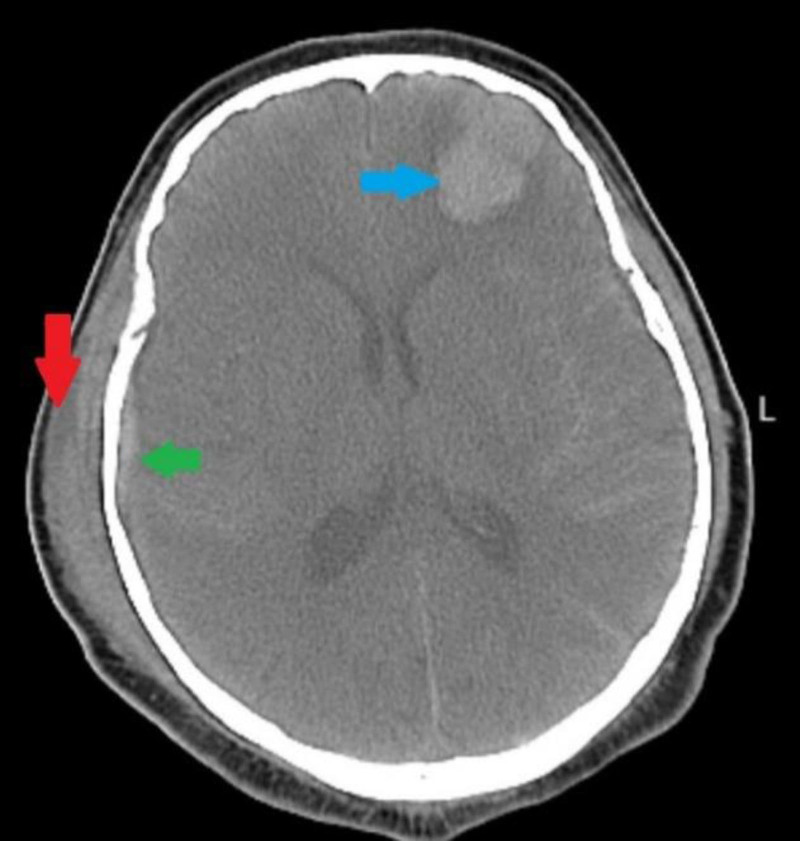
An axial non-contrast head CT image shows a right parietal epidural hematoma measuring approximately 7 cm × 2.5 cm (red arrow), accompanied by an underlying linear, minimally displaced skull fracture and pneumocephalus (green arrow). Bilateral diffuse subarachnoid hemorrhage is evident, alongside a small left tentorial subdural hematoma measuring 0.5 cm × 4 cm (blue arrow). Notably, there is no observable midline shift, indicating no significant mass effect. CT = computed tomography.

A 57-year-old male presented to our Emergency Department following a fall from 2 steps while ascending stairs. Upon arrival, the patient appeared dizzy, with no neck collar in place. His airway was intact, and his breathing effort was reduced but adequate, with a blood pressure of 100/70 mm Hg, heart rate of 100 bpm, oxygen saturation of 99%, and a Glasgow Coma Scale of 11/15. Pupils were equal, round, and reactive bilaterally. Initial management included applying a neck collar, securing intravenous (IV) access, initiating IV fluids, and providing oxygen support via a facial mask. An electrocardiogram showed no acute changes. A brain computed tomography (CT) revealed a linear, non-displaced fracture in the occipital skull, diffuse subarachnoid hemorrhage, a right-sided epidural hematoma, and a subdural hematoma and C3 vertebral body fracture as shown in Figure 1, and the neurosurgery team was immediately consulted. On examination, no focal neurological deficits were noted, though confusion and occipital tenderness were present.

The rest of the examination was normal. The patient has a medical history of diabetes mellitus, hypertension, ischemic heart disease (with the most recent stent placement 2 months ago), hypothyroidism, and rheumatoid arthritis. There is no history of previous surgeries, and he denies any known allergies. His current medications include Aspirin, Levothyroxine, insulin (administered twice daily), and Sitagliptin (DPP-4 inhibitor).

The patient was referred to our surgical-intensive care unit (ICU) for observation. On the next day, he was noncooperative so his physical findings weren’t perfectly obvious, but he was diagnosed with EuDKA based on his laboratory findings. Despite a blood glucose level of 134 mg/dL, which falls within normal limits, the patient exhibited hallmark features of DKA, including severe metabolic acidosis with a pH of 7.16, a critically low bicarbonate (HCO₃⁻) level of 2.8 mmol/L, and a significantly elevated anion gap of 24.9 mmol/L. These findings, coupled with the presence of respiratory alkalosis (partial pressure of carbon dioxide at 7.9 mm Hg) as shown in Table 1, likely reflect compensatory hyperventilation (Kussmaul respiration), and positive ketones in his urine analysis further supporting the diagnosis. The absence of marked hyperglycemia suggests a euglycemic variant of DKA, often associated with factors such as DPP-4 inhibitor use and extensive head trauma as in this case.

**Table 1:** Laboratory findings.

Labs	Normal ranges	Day 1	Day 4
*CBC*			
Hct	40–52%	33.78%	30.36%
Hb%	14–18 g/dL	10.49 g/dL	9.87 g/dL
MCH	27–31 pg/cell	24.88 pg/cell	24.89 pg/cell
MCHC	32–36 g/dL	31.05 g/dL	32.53 g/dL
MCV	82–94 fL	79.87 fL	76.51 fL
PLTs	150–400 × 10^3^/µL	159.9 × 10^3^/µL	238 × 10^3^/µL
RBC	4.6–6.2 × 10^12^/L	4.23 × 10^12^/L	3.96 × 10^12^/L
WBC	(5.0–10.0) × 10^9^/L	7.48 × 10^9^/L	4.96 × 10^9^/L
Neutrophils	45–65 cell/µL	69.4 cell/µL	51.9 cell/µL
Lymph	25–45 cell/µL	16 cell/µL	35.9 cell/µL
Monocytes	0–6	12.7	9.53
Eosinophils	0–2	0.063	1.5
Basophils	0–1	0.776	1.1
RDW	11.5–13.5%	15.2%	15.26%
ABS Neutrophils	2.5–7.5 × 10^9^/L	5.92 × 10^9^/L	2.577 × 10^9^/L
ABS Lymphocytes	1.5–3.5 × 10^9^/L	1.36 × 10^9^/L	1.78 × 10^9^/L
ABS Monocytes	0.04–0.8 × 10^9^/L	1.084 × 10^9^/L	0.74 × 10^9^/L
ABS Eosinophils	0.04–0.44 × 10^9^/L	0.005 × 10^9^/L	0.075 × 10^9^/L
ABS Basophils	0.015 × 10^9^/L	0.022 × 10^9^/L	0.055 × 10^9^/L
*Urine analysis*			
Protein	Nil	Nil	Nil
pH	Acid	Acid	Acid
Suger	Negative	+++	+++
Ketones	Negative	+++	Negative
Leukocytes	2–5 WBC/hpf	1–2 WBC/hpf	3–5 WBC/hpf
Erythrocytes	<4 cells/hpf	6–8 cells/hpf	8–10 cells/hpf
*Electrolytes*			
Na^+^	135–145 mEq/L	142 mEq/L	137 mEq/L
K^+^	3.6–5.2 mmol/L	4.27 mmol/L	4.2 mmol/L
Cl^-^	96–106 mmol/L	122.4 mmol/L	108.3 mmol/L
Phosphorus	2.5–4.5 mg/dL	2.2 mg/dL	4.1 mg/dL
*ABGs*			
pH	7.35–7.45	7.16	7.5
pCO_2_	35–45 mm Hg	7.9 mm Hg	23.1 mm Hg
pO_2_	30–55 mm Hg	77.7 mm Hg	90.9 mm Hg
HCO_3_	22–27 mmol/L	2.8 mmol/L	18.3 mmol/L
sO_2_	95%	92 %	98 %
Anion gap	4–14 mmol/L	24.9 mmol/L	14.4 mmol/L
Random blood sugar	70–140 mg/dL	134 mg/dL	144 mg/dL
CRP titer	Up to 6 mL/L	238.9 mL/L	42.3 mL/L
Osmolarity, serum	280–298 osmols/L	293.2 osmols/L	288 osmols/L
Osmolarity, urine	350–1300 osmols/L	577 osmols/L	388 osmols/L

ABG = arterial blood gas, pCO2 = partial pressure of carbon dioxide.

Prompt management was initiated to stabilize the patient and address metabolic derangements, with a focus on aggressive fluid resuscitation, insulin therapy, dextrose administration, discontinue Sitagliptin, and electrolyte correction. The total fluid volume administered was based on the patient’s body weight and clinical presentation, with an initial bolus of 1 to 1.5 L of normal saline given over the first hour, followed by maintenance fluids (10% dextrose saline) adjusted to the patient’s hydration status and kidney function. Insulin therapy was initiated via a continuous IV infusion, typically starting at 0.05 units/kg/hr, aiming to reduce blood glucose levels gradually (no more than 50–75 mg/dL per hour) to avoid complications like cerebral edema, with frequent blood glucose monitoring guiding adjustments. Electrolytes were corrected as follows: sodium was normalized with normal saline, potassium levels were closely monitored due to potential drops with insulin therapy, requiring supplementation (5–40 mEq/h) in the central venous catheter line according to Potassium level, and bicarbonate was corrected spontaneously.

After 4 days in the surgical ICU, the patient’s arterial blood gas analysis demonstrates significant improvement, with a pH of 7.5, partial pressure of carbon dioxide of 23.1 mm Hg, and bicarbonate (HCO₃⁻) of 18.3 mmol/L as shown in Table 1, indicating full recovery from severe metabolic acidosis. The patient showed gradual improvement in neurological function after the interventions. Upon initial assessment, there was evidence of altered mental status and reduced cognitive function, likely related to the metabolic disturbances. After addressing metabolic abnormalities and management, the patient exhibited better orientation and responsiveness.

For his head trauma and given the absence of midline shift, significant mass effect, or evidence of increased intracranial pressure, a decision was made for *conservative neurosurgical management* with close neurological monitoring in the ICU. Serial neurological assessments and repeat CT imaging were performed to monitor the evolution of the hematomas.

The epidural and subdural hematomas were small and stable in size, and surgical intervention was not deemed necessary. The diffuse subarachnoid hemorrhage was managed supportively with strict blood pressure control and close observation for vasospasm.

For the C3 vertebral body fracture, a spine surgery consultation was obtained. Since the fracture was non-displaced and the patient exhibited no focal neurological deficits, *cervical spine immobilization* was implemented using a hard cervical collar.

Antiepileptic prophylaxis was initiated with *levetiracetam* to reduce the risk of post-traumatic seizures. Intracranial pressure was monitored clinically, and mannitol was not required. The patient remained hemodynamically stable, and no signs of neurological deterioration were observed throughout the ICU stay.

**Figure 2.**
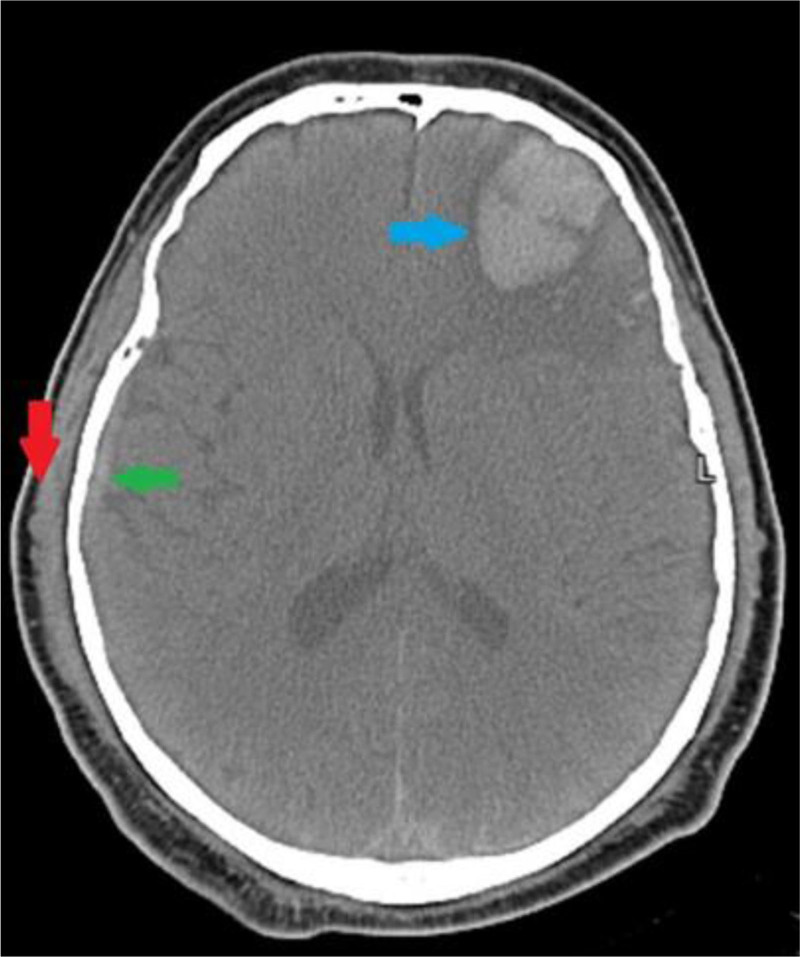
A follow-up non-contrast head CT for the same patient, demonstrating significant interval improvement. The right parietal epidural hematoma has markedly decreased in size, with associated resolution of the pneumocephalus (green arrow). The linear skull fracture remains visible but without evidence of complication. The bilateral subarachnoid hemorrhage is less conspicuous, and the left tentorial subdural hematoma (blue arrow) shows no progression in size. Importantly, there is no evidence of midline shift or significant mass effect. CT = computed tomography.

Follow-up tests were conducted to assess both neurological and metabolic recovery. A CT scan has been performed daily to rule out structural brain lesions or ischemic events, especially given the patient’s history of ischemic heart disease with some improvement signs as shown in Figure 2, which was taken on day 4. Blood tests, including glucose, electrolytes, ketones, and arterial blood gas, were monitored regularly to track improvements in metabolic parameters and acid-base status with continuously normal values.

Despite the patient’s complex medical history, including ischemic heart disease and rheumatoid arthritis, he achieved full recovery and his follow up after 1 week, 1 month, and 2 months was good with full recovery from the event.

Differential diagnoses for metabolic acidosis include DKA, alcoholic ketoacidosis, starvation ketosis, and lactic acidosis. Lactic acidosis should be considered, particularly if the patient has sepsis, shock, or signs of poor perfusion, while DKA or other forms of ketoacidosis may result from insulin deficiency or metabolic stress. The patient’s altered mental status could be attributed to metabolic acidosis, hypoglycemia, or other acute systemic conditions such as sepsis, uremia, or liver failure. In patients with a history of ischemic heart disease, cerebral hypoperfusion may also contribute to altered sensorium. Toxicological causes, such as drug overdose or alcohol intoxication, should be considered, particularly if there are signs of physical trauma or an unclear medical history. Physical trauma itself can complicate the clinical presentation by introducing factors such as hemorrhagic shock, sepsis, or acute pain syndromes, all of which can exacerbate metabolic disturbances and contribute to altered mental status.

This case highlights the importance of recognizing euglycemic DKA in trauma patients, especially those on medications like DPP-4 inhibitors, and the need for close monitoring and early intervention in such cases. This progress reflects the effectiveness of our timely and precise diagnosis, along with the early initiation of an individualized management plan. Our proactive approach ensured the administration of essential interventions, with fluid resuscitation and insulin administration playing a pivotal role in stabilizing the patient’s condition and reversing the metabolic derangements. The remarkable recovery underscores the importance of early recognition and swift management in critical cases, as well as the dedication and teamwork demonstrated throughout this case. The patient’s current state – more cooperative, conscious, and well – serves as a testament to the success of these efforts.

## 3. Discussion

DKA is a potentially life-threatening complication of diabetes. Euglycemic DKA (EuDKA) is a rare variant, accounting for 2.6% to 3.2% of DKA admissions being normoglycemic, a condition that primarily occurs in type 1 diabetes, though it can occasionally be seen in type 2 diabetes. The exact mechanism of euglycemic DKA remains unclear, but it has been linked to factors such as DPP-4 inhibitors, SGLT-2 inhibitors, carbohydrate restriction, alcohol consumption, and pregnancy.^[5]^ Among these, DPP-4 inhibitors are emerging as a possible precipitant, though research remains limited.

However, in normal cases, the primary mechanism of action for DPP-4 inhibitors involves the inhibition of DPP-4 activity in peripheral plasma, preventing the inactivation of the incretin hormone glucagon-like peptide-1. This leads to increased levels of circulating intact glucagon-like peptide-1, which stimulates insulin secretion and inhibits glucagon release. As a result, glucose utilization is enhanced, and hepatic glucose production is reduced, ultimately lowering both postprandial and fasting glucose levels, thereby decreasing HbA1c.^[6]^ However, there is limited literature exploring the link between EuDKA and DPP-4 inhibitors, but this report suggests the development of traumatic brain injury (TBI) before presentation. The pathophysiological mechanism by which DPP-4 inhibitors contribute to EuDKA remains under investigation. It has been suggested that these drugs may alter the normal counter-regulatory hormonal response to insulin deficiency, particularly during periods of metabolic stress, leading to reduced glucose utilization and ketone production.^[7]^ Additionally, DPP-4 inhibitors may impair insulin secretion and promote glucagon release under stressful conditions such as head trauma, exacerbating the development of ketoacidosis despite normal or low blood glucose levels.

Diagnosing DDP-4 inhibitors-induced EuDKA can be challenging, especially when critical information about the pathophysiology is hard to obtain due to TBI.^[8]^ Standard diagnostic criteria for DKA include a plasma glucose level > 250 mg/dL, positive urinary or serum ketones, arterial pH < 7.3, serum bicarbonate < 18 mEq/L, and a high anion gap. While hyperglycemia is central to the diagnosis, DKA can occasionally present with normal glucose levels, a condition known as EuDKA, which may be overlooked.^[8]^ Thus, a high index of suspicion is required for diagnosis, especially in the context of polypharmacy and complex comorbidities. We present a case of EuDKA in a patient with a blood sugar level of 134 mg/dL. DPP-4 inhibitor was identified as the most likely precipitating factor in this case in addition to the head trauma.^[9]^

As the diagnosis confirmed, volume resuscitation should be the focus in the initial management of EuDKA. Fluid loss in EuDKA can range from 6 to 9 L, making rehydration crucial for restoring adequate tissue perfusion and correcting metabolic abnormalities. The American Diabetes Association recommends administering 1 to 1.5 L per hour of normal saline or lactated Ringer solution during the first 1 to 2 hours of fluid resuscitation. IV fluid supplementation should continue based on the patient’s condition until the anion gap closes and acidosis resolves. Monitoring of ketones and electrolytes is essential, with ketones measured hourly and electrolytes every 2 hours, until blood ketone levels drop below 0.6 mmol/L and electrolytes stabilize.^[10]^

Despite the absence of hyperglycemia in EuDKA, insulin remains a critical component of treatment. Insulin helps suppress ketone formation by promoting glucose utilization and decreasing gluconeogenesis and glycogenolysis. After adequate fluid replacement, a continuous insulin infusion should be initiated at a rate of 0.05 U/kg/hour to 0.1 U/kg/hour, provided serum potassium levels are above 3.3 mEq/L. Since insulin facilitates the movement of potassium into muscle cells, insulin therapy should be postponed if hypokalemia is present until potassium levels are normalized. Potassium levels should be closely monitored every 2 hours until they stabilize. Once EuDKA is resolved, the patient can transition to subcutaneous long-acting insulin and premeal rapid-acting insulin to manage blood glucose levels. The insulin infusion should continue for at least 1 hour after administering subcutaneous insulin.^[10,11]^

In addition, the treatment of EuDKA requires the addition of dextrose 5% (D5W) to fluids due to blood glucose concentrations being < 250 mg/dL. Dextrose is necessary to restore normal cellular glucose utilization, which in turn enhances ketone body clearance and reduces their production. Additionally, the inclusion of D5W in fluids helps prevent hypoglycemia by providing an exogenous glucose source while insulin is being used. If ketoacidosis persists despite the administration of D5W, dextrose 10% may be considered as an alternative. Along with addressing the underlying cause which was the head trauma in this case.^[12]^

In trauma patients, such as the case presented here, identifying EuDKA is crucial for appropriate management. Discontinuing the DPP-4 inhibitor, in this case, sitagliptin, is necessary upon diagnosis to prevent further exacerbation of ketoacidosis. The role of other factors, such as head trauma, in precipitating EuDKA must also be considered to ensure comprehensive care.

Given the life-threatening nature of EuDKA, early recognition and prompt treatment are essential. This case highlights the importance of monitoring patients on DPP-4 inhibitors closely for signs of EuDKA, particularly following trauma. Further research into the mechanisms and optimal management strategies for EuDKA in the context of newer antidiabetic therapies is warranted.

## 4. Conclusion

This was a unique case of euglycemic DKA following TBI, in which the initial diagnosis was delayed. EuDKA is a rare but potentially life-threatening condition that can be triggered by factors such as DPP-4 inhibitors, particularly in patients with trauma. The absence of hyperglycemia in EuDKA can complicate diagnosis, emphasizing the importance of careful monitoring and consideration of this condition, especially in trauma patients using medications like DPP-4 inhibitors. Early identification and prompt treatment are essential, including discontinuation of the precipitating agent, volume resuscitation, and insulin therapy to manage ketosis and metabolic abnormalities. Close monitoring of electrolytes, ketones, and blood glucose levels is critical for effective management. This case highlights the need for heightened awareness and a multidisciplinary approach to diagnosing and managing EuDKA, ensuring that both the underlying causes and metabolic disturbances are addressed for optimal patient outcomes.

## Acknowledgments

We would like to express our gratitude to Al-Ahli Hospital and its SICU team for their invaluable support and collaboration.

## Author contributions

**Conceptualization:** Mohammed Seyaj, Malek Burqan.

**Data curation:** Kenana Altell, Razan Sobeih, Lara Asafrah, Malek Burqan, Mohammed Shahateet.

**Formal analysis:** Kenana Altell, Malek Burqan.

**Investigation:** Isam Shammas, Mohammed Seyaj, Malek Burqan, Mamoun Abuhalawa, Mohammed Shahateet, Saeed Itkaidek.

**Methodology:** Isam Shammas, Mohammed Shahateet.

**Project administration:** Kenana Altell.

**Resources:** Lara Asafrah.

**Software:** Mohammed Shahateet.

**Supervision:** Isam Shammas, Mohammed Seyaj.

**Validation:** Isam Shammas, Mohammed Seyaj, Mamoun Abuhalawa, Saeed Itkaidek.

**Visualization:** Isam Shammas, Mamoun Abuhalawa.

**Writing – original draft:** Kenana Altell, Razan Sobeih, Lara Asafrah, Saeed Itkaidek.

**Writing – review & editing:** Isam Shammas, Mohammed Seyaj, Kenana Altell, Razan Sobeih.
